# Synthesis and Photophysical Properties of Tetra- and Octasubstituted Phosphorous Oxide Triazatetrabenzcorrole Photosensitizers

**DOI:** 10.1155/2008/498916

**Published:** 2008-03-03

**Authors:** Edith M. Antunes, Tebello Nyokong

**Affiliations:** Department of Chemistry, Rhodes University, Grahamstown 6140, South Africa

## Abstract

The synthesis of phosphorous oxide triazatetrabenzcorroles (TBC) tetra- (**9**, **11**) or octa- (**13**) substituted on the ring with halogenated functional groups is reported. The complexes are not aggregated in dimethylsulfoxide (DMSO) and show solubility in solvents such as pyridine. The *Q* band absorption spectra of the complexes are red-shifted compared to unsubstituted PTBC. The latter complex shows a large triplet lifetime (1.7 milliseconds), higher than for MPc derivatives. The chlorinated derivatives show good triplet yields (Φ*_T_*∼ 0.46 and 0.36) and relatively long lifetimes (256 and 452 microseconds), respectively, for **11** and **13**.

## 1. INTRODUCTION

The immense and diverse potential of phthalocyanines (Pc) in a variety of technical (chemical sensors [1], liquid crystals [2], electrocatalysis [3], and nonlinear optics [4]) and medicinal (primarily photodynamic therapy [5–8]) applications has generated a great deal of interest in these macrocyclic compounds. This, together with the extraordinary stability of these complexes, has resulted in considerable research being carried out on the phthalocyanine complex upon incorporation of nearly all metals in the periodic table into the Pc core. Complexes with metalloids and nonmetals of Groups IVA and VA are of particular interest due to the two different valence/oxidation states available to the central atom.

**Scheme 1:**
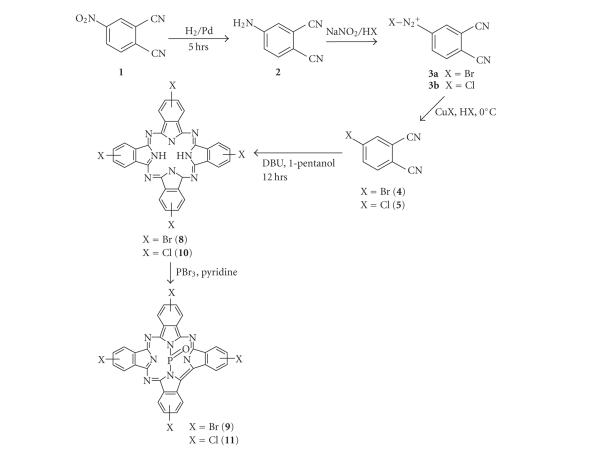
Synthetic procedure for the tetrasubstituted PTBC complexes.

**Scheme 2:**
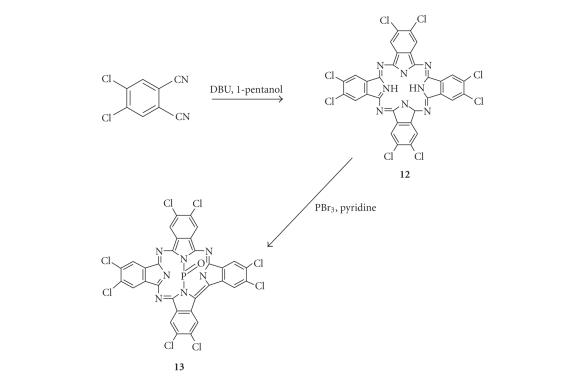
Synthesis of the octachlorosubstituted PTBC (**13**).

Triazatetrabenzcorroles (TBC) are phthalocyanine-like compounds which have lost one of the bridging nitrogen atoms [9]. The synthesis of Ge, Si, Ga, and Al TBC complexes have been reported [10, 11]. We have recently reported on a microwave synthesis of a sulfonated SnTBC [12]. Gouterman et al. were the first to report on the synthesis and unusual electronic spectra of the PcP^III^ compared to PcP^V^ [9, 13]. It became apparent that upon complexation with the trivalent phosphorous, a bridging nitrogen was lost to form the phosphorous oxide tetrabenzcorroles (PTBC). Since then, a number of octa- and tetrasubstituted PTBC derivatives have been synthesized [14–19]. Most complexes synthesized contained alkyl chain ring substituents. High fluorescence quantum yields [18] and singlet oxygen quantum yields [15] have been reported for the complexes. The water-soluble tetrasulphonated PTBC showed good photodynamic therapy towards HeLa cells [19]. For applications in PDT, high triplet lifetimes and yields are desired; however there are no reports on the triplet-state behavior of the PTBC complexes. In this work, we report on PTBC complexes tetra- or octasubstituted with chloride and bromide ring substituents (see Schemes 1 and 2), since the halogens are expected to enhance intersystem crossing, resulting in high triplet yields due to the heavy atom effect. A PTBC derivative containing a mixture of butoxy and chloro ring substituents has been reported [17], but no photophysical data has been reported for halogenated PTBCs.

## 2. EXPERIMENTAL PROCEDURES

**Materials**

Dimethylsulfoxide (DMSO), methanol (MeOH), phosphorous tribromide (PBr_3_), deuterated chloroform (CDCl_3_), deuterated dimethylsulfoxide (DMSO-*d*_6_), deuterated pyridine (Pyr-*d*_5_), 4,5 dichlorophthalonitrile, 1,8-diazabicyclo undec-7-ene (DBU), 4-nitrophthalonitrile, copper (I) bromide, copper (I) chloride, hydrobromic acid, hydrochloric acid, palladium on carbon (Pd/C), and dicyanobenzene were purchased from Sigma-Aldrich (Miss, USA) and used as received. Pyridine and 1-pentanol were obtained from Sigma-Aldrich and dried prior to use. Column chromatography was performed on silica gel 60 (0.04–0.063 mm).

**Equipment**

Ground-state electronic absorption spectra were recorded on a Varian Cary 500 UV-Vis-NIR spectrophotometer. ^1^H and ^13^C NMR spectra were obtained using a Bruker AMX 400 MHz and a Bruker Avance II+ 600 MHz NMR spectrometer. Fluorescence emission and excitation spectra were recorded on a Varian Cary Eclipse spectrofluorimeter, while FT-IR spectra (KBr pellets) were recorded on a Perkin-Elmer spectrum 2000 FT-IR spectrometer. MS data was recorded on a Shimadzu KRATOS Maldi MS instrument.

Triplet absorption and decay kinetics were recorded on a laser flash photolysis system, the excitation pulses were produced by an Nd: YAG laser (Quanta-Ray, 1.5 J/90 ns) pumping a dye laser (Lambda Physic FL 3002, Pyridin 1 in methanol). The analyzing beam source was from a Thermo Oriel xenon arc lamp, and a photomultiplier tube was used as detector. Signals were recorded with a two-channel digital real-time oscilloscope (Tektronix TDS 360) where the kinetic curves were averaged over 256 laser pulses. Triplet lifetimes were determined by exponential fitting of the kinetic curves using OriginPro 7.5 software. Solutions for triplet yield and lifetime determinations were degassed with argon before use.

### 2.1. Syntheses and characterization

4,5-Dichlorophthalonitrile and dicyanobenzene were commercially obtained. Unsubstituted PTBC (**7**) was synthesized from unmetallated phthalocyanine (**6**) and PBr_3_ in a 16.4% yield, and characterized as reported in the literature [9, 13, 19]. 4-Aminophthalonitrile (**2**) and 4-bromophthalonitrile (**4**) were synthesised following literature procedures [20, 21] with some minor modifications. 4-Chlorophthalonitrile (**5**) was synthesised by modifying the procedure used for compound **4**.

#### 2.1.1. Preparation of 4-aminophthalonitrile (2)

4-Nitrophthalonitrile(**1**) (1.00 g, 5.78 mmol) was placed in a round bottom flask and 100 mL of ethanol added to obtain a suspension. The catalyst Pd/C (55 mg) was added to the flask, the apparatus evacuated and then filled with hydrogen and the mixture vigorously stirred at room temperature until the absorption of hydrogen had completely stopped. The reaction mixture was subsequently filtered over celite and the solution evaporated in vacuo. Yield: 98%. ^1^H NMR (400 MHz, DMSO-*d*6) *δ* ppm 7.39 (dd, *J =* 8.66, 1.34 Hz, 1H), 6.93 (d, *J =* 2.19 Hz, 1H), 6.81 (dd, *J =* 8.67, 2.18 Hz, 1H), 6.36 (br s, 2H).

#### 2.1.2. Preparation of 4-bromophthalonitrile (4)

Compound **2** (500 mg, 3.5 mmol) was taken up in a mixture of water (4 mL) and hydrobromic acid (4 mL, 48%) and the solution cooled to 0°C using an ice-salt bath. A solution of sodium nitrite (276 mg, 4 mmol) in water (2 mL) was then added dropwise to the acid mixture to form the diazonium salt **3a** as an intermediate. Copper (I) bromide (1.00 g, 6.97 mmol) was dissolved in HBr (4 mL, 48%) and cooled to 0°C. The cold diazonium salt solution was then added dropwise to the CuBr reagent and the solution stirred for 1 hr at 0°C After an hour, the solution was left to stand at room temperature overnight. The aqueous solution was then extracted with ethyl acetate (3 × 15 mL), the combined organic extracts washed with brine, dried over magnesium sulphate and concentrated to give 248 mg of **4**. Yield: 34%. ^1^H NMR (400 MHz, CDCl_3_) *δ* ppm 7.96 (d, *J =* 1.78 Hz, 1H), 7.89 (dd, *J =* 8.37, 1.89 Hz, 1H), 7.68 (d, *J =* 8.37 Hz, 1H). ^13^C NMR (75 MHz, CDCl_3_) *δ* ppm 136.6 (d), 136.3 (d), 134.4 (d), 128.1 (s), 117.3 (s), 114.7 (s), 114.5 (s), 114.0 (s).

#### 2.1.3. Preparation of 4-chlorophthalonitrile (5)

A similar procedure to the one above was used to prepare compound **5**. Compound **2** (500 mg, 3.5 mmol) was dissolved in a water (4 mL)/hydrochloric acid (5 mL, 32%) mixture, and the sodium nitrite (276 mg, 4 mmol) in water (2 mL) reagent was added to the amine hydrochloride solution to form the diazonium salt (**3b**). Copper (I) chloride (1.00 g, 10.1 mmol) was dissolved in HCl (5 mL) and cooled to 0°C. The diazonium salt was then added to the CuCl reagent and treated in the same manner as reported above. Compound **5** was thus produced in a 58% yield. ^1^H NMR (400 MHz, CDCl_3_) *δ* ppm 7.80 (d, *J =* 1.88 Hz, 1H), 7.75 (s, 1H), 7.73 (d, *J =* 1.87 Hz, 1H). ^13^C NMR (75 MHz, CDCl_3_) *δ* ppm 140.2 (s), 134.5 (d), 133.7 (d), 133.5 (d), 117.4 (s), 114.6 (s), 114.2 (s), 114.1 (s).

#### 2.1.4. Preparation of tetrabromo phosphorous oxide triazatetrabenzcorrole (9, PTBrTBC)

Complex **9** was synthesized from the unmetallated derivative (**8**). The first step was the synthesis of **8**, following established methods [22] as follows: **4** (200 mg, 0.97 mmol) was reacted with DBU (0.36 mL) in 1-pentanol under reflux for 12 hours under a nitrogen atmosphere. The dark green mixture was cooled down to room temperature, then methanol (5 mL) and water (2 mL) were added and the mixture precipitated out and centrifuged. This green solid complex **8**, confirmed to be a phthalocyanine molecule by its UV spectrum, was not purified any further and was employed as is for the synthesis of complex **9**. Complex **9** was synthesized by heating (under reflux) a mixture of complex **8** (100 mg, 0.12 mmol) and PBr_3_(0.34 mL, 3.60 mmol) in pyridine at 90 to 100^°^C for 2 hours. The reaction was then allowed to cool to room temperature and poured carefully into water and allowed to stand overnight. The green product obtained was then centrifuged and washed with copious amounts of water. Upon drying, the precipitate was chromatographed on a silica gel column using pyridine as the eluant. The complex was collected as a dark green band, while the unmetallated Pc remained at the top of the column. Yield: 13.5%. UV/Vis (DMSO), *λ* *_max_*⁡nm (log⁡*ε*)]: 662 (4.86), 630 (4.56), 602 (4.29), 578 (3.79), 448 (5.19), 439 (4.94), 418 (4.70). [(KBr) *v* *_max_*⁡/cm^−^^1^]: 3063, 1717, 1608, 1506, 1451, 1396, 1326, 1298 (P=O), 1271, 1151, 1109, 1077, 1043, 967, 956, 832, 813, 758, 723, 691, 578. ^1^H NMR (600 MHz, Pyr-*d*_5_) *δ* ppm 10.15–9.92 (m, 4H), 9.83–9.57 (m, 4H), 8.59–8.41 (m, 4H). ^31^P NMR (162 MHz, Pyr-*d*_5_) *δ* ppm –198.6. MALDI *m*/*z*: Calc. 860.8, found 861.1 {Br_4_(TBC)P(O)}.

#### 2.1.5. Preparation of tetrachloro phosphorous oxide triazatetrabenzcorrole (11, PTClTBC)

Complex **11** was synthesized as explained above for **9**, except that complex **5** (200 mg, 1.23 mmol) was reacted with DBU (0.24 mL) in 1-pentanol instead of compound **4** to obtain the 4-chloro H_2_Pc analogue (complex **10**). The presence of the metal-free Pc was again confirmed by its UV spectrum. Complex **10** was then employed for the synthesis of complex **11** (100 mg, 0.15 mmol) by reacting it with PBr_3_(0.42 mL, 4.50 mmol) in hot pyridine. Yield: 27.7%. UV/Vis (DMSO), *λ*_max_⁡/nm (log⁡*_ε_*)]: 661 (4.56), 630 (4.26), 603 (3.99), 447 (4.82), 438 (4.59), 417 (4.39). [(KBr) *v*_max_⁡/cm^−^^1^]: 2922, 1717, 1609, 1505, 1456, 1399, 1328, 1297 (P=O), 1143, 1068, 973, 910, 814, 769, 724, 690, 580. ^1^H NMR (600 MHz, Pyr-*d*_5_) *δ* ppm 9.97–9.66 (m, 8H), 8.45–8.26 (m, 4H). ^31^P NMR (162 MHz, Pyr-*d*_5_) *δ* ppm –198.5. MALDI *m*/*z*: Calc. 682.9, found 682.0 {Cl_4_(TBC)P(O)}.

#### 2.1.6. Preparation of octachloro phosphorous oxide triazatetrabenzcorrole (13, POClPBC)

The first step was the synthesis of metal-free complex **12**, using the same procedure as outlined above for the synthesis of **8**, except that 4,5-dichlorophthalonitrile (300 mg 1.53 mmol) was reacted with DBU (0.12 mL) instead of complex **4**. Confirmation of the formation of the H_2_Pc (**12**) was provided by the UV spectrum. Complex **13** was then synthesized as described for complexes **9** and **11** using **12** (100 mg, 0.128 mmol) and PBr_3_ (0.36 mL, 3.84 mmol). A paucity of proton signals was expected and observed in the ^1^H NMR spectrum of this octasubstituted complex. Yield: 8.6%. UV/Vis (DMSO), *λ*_max_⁡/nm (log⁡*ε*)]: 667 (3.47), 635 (3.19), 606 (2.94), 452 (3.83), 442 (3.61), 422 (3.46), 411 (3.42). [(KBr) *v*_max_⁡/cm^−^^1^]: 2927, 1726, 1586, 1521, 1349, 1298 (P=O), 1248, 1197, 1138, 948, 908, 837, 743, 602. ^1^H NMR (600 MHz, Pyr-*d*_5_) *δ* ppm 8.07 (m, 8H). ^31^P NMR (162 MHz, Pyr-*d*_5_) *δ* ppm −197.4. MALDI *m*/*z*: not observed.

### 2.2. Photophysical and photochemical parameters

#### 2.2.1. Fluorescence quantum yields and lifetimes

The comparative method was used to determine the fluorescence quantum yields (Φ*F*) according to the following equation [23], utilizing unsubstituted ZnPc in DMSO as the standard (Φ*F* = 0.18) [24]:

(1) $$\Phi_{F}=\Phi_{F\left(\text{Std }\right)}\frac{F\cdot \;A_{\text{Std }}\;\cdot \;\eta^{2}}{F_{\text{Std}}\cdot \,\;\,\;A\,\;\cdot \eta_{\text{Std}}^{2}},$$

where *F* and *F*_Std_ are the areas under the fluorescence curve of the sample and the standard, respectively. Similarly, *A* and *A*_Std_ are the absorbance of the compound and the standard at the excitation wavelength, *η* and *η*_Std_ are the refractive indices of solvents used for the sample and the standard, respectively.

Natural or radiative lifetimes (*τ*_N_) were estimated using PhotochemCAD program which uses the Strickler-Berg equation [25]. The fluorescence lifetimes (τ*_F_*) were evaluated using the following equation:

(2) $$\Phi_{F}=\frac{\tau_{F}}{\tau_{N}}.$$

The rate constants for intersystem crossing from the excited singlet state to the triplet state (k_ISC(S−T)_) were estimated using the following equation [26]:

(3) $${\text{k}}_{\text{ISC}\left(\text{S}-\text{T}\right)}=\left(1/\tau_{f}\right)-\left(1/\tau_{f^{0}}^{}\right),$$

where *τ*_f_ and *τ*_f_^0^ are the excited singlet-state lifetimes for the halogenated derivatives and unsubstituted PTBC, respectively. Similarly, the rate constants for intersystem crossing from the triplet state to the ground state (k_ISC__(T−S))_ were estimated using the following equation:

(4) $${\text{k}}_{\text{ISC}\left(\text{T}-\text{S}\right)}=\left(1/\tau_{T}\right)-(1/\tau_{T^{0}}),$$

where *τ* *_T_* and *τ* *_T_*_0_ are the excited triplet-state lifetime for the halogenated derivatives and unsubstituted PTBC, respectively.

#### 2.2.2. Triplet quantum yields and lifetimes

Solutions of the PTBC complexes were bubbled with argon in a 1 cm pathlength spectrophotometric cell, irradiated at the *Q* band of the respective PTBC complexes, with the triplet quantum yields (Φ*_T_*) determined by the triplet absorption method. The comparative method [27] was applied as in the following equation, using ZnPc in DMSO as the standard:

(5) $$\Phi_{T}={\Phi_{T}^{\text{std}}}_{}\cdot \frac{\Delta {\text{A}}_{T}\cdot \,\;\varepsilon_{T}^{\text{Std}}}{\Delta {{\text{A}}_{T}^{\text{Std}}\cdot \;\varepsilon_{T}}_{}}.$$

Changes in the triplet-state absorbances of the PTBC derivative and the standard are represented by *Δ* *A* *_T_* and $\Delta {{\text{A}}_{T}^{\text{Std}}}_{}$, respectively; while *ε* *_T_* and $\varepsilon_{T}^{\text{std}}$ are the triplet-state molar extinction coefficients for the PTBC derivative and the standard, respectively; while $\Phi_{T}^{\text{std}}$ is the triplet quantum yield for the standard (Φ*_T_* = 0.65 for ZnPc in DMSO) [28]. Triplet lifetimes (*τ* *T*) were determined by exponential fitting of the kinetic curves using OriginPro 7.5 software.

Quantum yields of internal conversion (Φ_IC_) were obtained from the following equation, which assumes that only three processes (fluorescence, intersystem crossing, and internal conversion) jointly deactivate the excited singlet state of PTBC derivatives:

(6) $$\Phi_{\text{IC}}=1-(\Phi_{F}+\Phi_{T}).$$

## 3. RESULTS AND DISCUSSION

### 3.1. Synthesis and characterization

Substituted phthalocyanines are generally prepared by cyclotetramerization of substituted phthalonitriles. 2(3), 9(10), 16(17), 23(24)-Tetrasubstituted phthalocyanines can be synthesized from 4-substituted phthalonitriles [22], while octasubstituted phthalocyanines can be synthesized from 4,5-dichlorophthalonitrile [29]. In the case of tetrasubstituted derivatives, a mixture of four possible structural isomers are obtained, which can be designated by their molecular symmetry as *C*_4_ *_h_*, *C*_2_ *_υ_*, *C* *_s_*, and D_2_ *_h_*. In this study, synthesized tetrasubstituted phthalocyanine compounds are obtained as isomer mixtures as expected. No attempt was made to separate the isomers of **9** and **11.**

A variety of halogenated phosphorous oxide triazatetrabenzcorroles (complexes **9**, **11,** and **13**) were prepared by treatment of unmetallated phthalocyanines with PBr_3_ in pyridine according to literature procedures [19]. The products thus obtained were then subjected to silica gel column chromatography using pyridine as an eluant.

Generally, phthalocyanine complexes are insoluble in most organic solvents; however introduction of substituents to the ring increases the solubility. The halogenated complexes (particularly **9** and **11**) exhibited excellent solubility in organic solvents such as pyridine and DMSO. For comparative purposes, the unsubstituted PTBC was synthesized and found to be soluble in DMSO, but only sparingly in pyridine.

The new compounds were characterized by UV-vis, IR, mass, and NMR spectroscopies (including ^31^P NMR) and the analyses were consistent with the predicted structures as shown in Section 2. However, mass spectral data proved to be difficult to obtain for complex **13**. The P=O vibrations were observed at *~*1295 cm^−1^(in accordance with [14]) in the IR spectra, confirming the presence of *O* coordinated to the phosphorous atom. This was corroborated by the ^31^P NMR shifts obtained, that is, *~* −198 ppm, which is typical of a P=O bond [14]. ^1^H NMR investigations of **9** and **11** gave the characteristic chemical shifts, with three proton signals integrating for a total of 12 for each complex. For complex **13**, a multiplet due to the nonperipheral protons, was observed in the ^1^H NMR spectrum.

**Figure 1:**
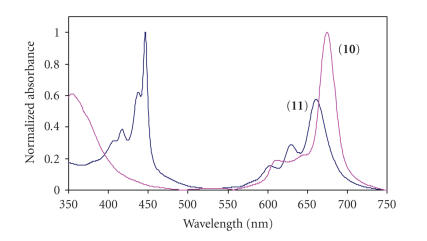
Spectrum of metal-free Pc (**10**) and transformation upon formation of PTClTBC (**11**) in DMSO. Concentration = 3.59 × 10^−5^ mol dm^−3^.

TBC complexes have distinct UV-Vis spectra with a sharp peak at *~*450 nm [16–19], which can be employed in their characterization. The formation of the TBC complexes occurs when the MPc molecules no longer retains a Pc moiety as they no longer have the fourth azomethine nitrogen (see Schemes 1 and 2). It is believed [11] that in the presence of excess metal halide, the bridge nitrogen of the Pc is eliminated, forming TBC. In this study, the unmetallated Pc derivatives (**8,** **10** and **12**) were formed first, which, upon reaction with PBr_3_, resulted in the formation of PTBC derivatives. This was judged spectroscopically by the collapse of the sharp *Q* band in the visible region of unmetallated Pcs to three bands (in the *Q* band region), together with the formation of the sharp Soret band at 440 nm (see Figure 1). The spectra of unmetallated Pcs **6**, **8**, **10,** and **12** in DMSO and pyridine showed a single *Q* band (see Figure 1) which is uncharacteristic of unmetallated Pcs. Typically, unmetallated Pcs show a split *Q* band due to lack of symmetry. Solvation in polar aprotic solvents (such as DMSO and pyridine) occurs through their unshared electrons. Thus, in DMSO (see Figure 1) and pyridine, the spectra of **8**, **10**, and **12** did not show the normal splitting of the *Q* band that is typical of free-base phthalocyanines, showing instead a single sharp *Q* band. The same applies to H_2_Pc in pyridine. The nonsplit *Q* band is a result of the basicity of the solvents. It has been documented that in strongly basic solvents, the inner pyrrole hydrogens are acidic enough to dissociate resulting in a charged system (Pc^−2^) which becomes symmetric and thus possesses an unsplit *Q* band [30].

**Figure 2:**
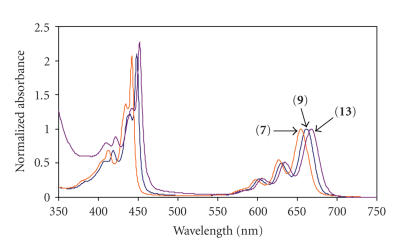
Comparison of the electronic spectra of various PTBC complexes synthesized in DMSO. Concentrations = 8.06 × 10^−5^ mol dm^−3^(**7**), 1.95 × 10^−5^ mol dm^−3^(**9**), 9.27 × 10^−5^ mol dm^−3^(**13**).

Figure 2 compares the spectra of the complexes synthesized in this work, while Table 1 lists the *Q* and *B* band maxima. It is clear in Table 1 and Figure 2 that the presence of bromines and chlorines shifts the spectra to the red region. The red shift of spectra on halogenation has been observed before [26] for ZnPc derivatives.

**Figure 3:**
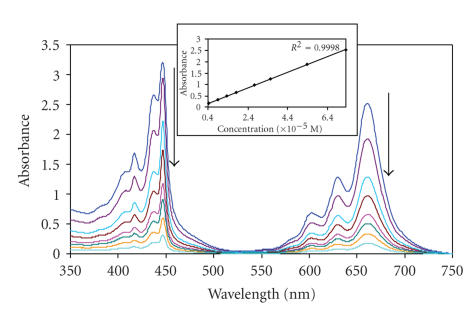
Absorption spectra of (**11**) in DMSO at different decreasing concentrations. Concentrations from 8 × 10^−5^ to 5 × 10^−6^ M. Inset: plot of absorbance versus concentration.

Aggregation in phthalocyanines and related complexes is usually depicted as a coplanar association of rings progressing from monomer to dimer and higher-order complexes. It is dependent upon the concentration of the complex, the nature of the solvent, as well as the nature of the substituents and the complexed metal ions. In this study, the aggregation behavior of the TBC complexes (**7**, **9**, **11**, and **13**) was investigated in DMSO (see Figure 3). The complexes did not show aggregation at concentrations less than 8 × 10^−^^5^ mol dm^−3^. A linear plot of absorbance versus concentration was obtained in this concentration range.

**Figure 4:**
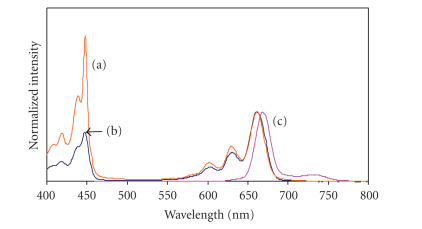
Comparison of the absorbance (a), excitation (b), and emission (c) spectra of complex **9**. Excitation wavelength = 380 nm.

The shapes of the excitation spectra for the TBC complexes were similar to the absorption spectra (see Figure 4). However, these spectra were not mirror images of the fluorescence spectra for the PTBC derivatives, in that the emission spectra showed only a single band, while the *Q* band of the absorption spectra has a split *Q* band. The observation of a single-emission band in the *Q* band region is typical of unsymmetric phthalocyanine complexes such as unmetallated derivatives [31–33]. Metal-free Pcs are known to fluoresce with only one main peak in non-aqueous media which has been assigned as the 0–0 transition of the fluorescence [31].

The emission spectra were slightly red-shifted with Stokes shifts ranging from 5 to 9 nm, suggesting no change in nuclear configurations following excitation. The largest shifts were observed for the POClTBC (**13**) and PTBrTBC (**9**), while the smallest shift was observed for the PTBC derivative (**7**).

### 3.2. Photophysical and photochemical studies

The fluorescence quantum yields (Φ*_F_*) of the PTBC derivatives are given in Table 2. These values are much lower than reported for MPc complexes, except for the unsubstituted PTBC complex, which gives Φ*_F_* values in the range for MPc complexes [34]. The low values obtained for the halogenated derivatives are most likely due to the heavy atom effect of the halide functional group, which encourages intersystem crossing to the triplet state. Halogenation of ZnPc [26] has been reported to give a remarkable decrease in fluorescence quantum yields and lifetimes, since incorporation of a halogen into the photosensitizer increases the level spin-orbit coupling.

Fluorescence lifetimes (*τ* *_F_*, Table 3) were calculated using the Strickler-Berg equation. Using this equation, a good correlation has been [35] found between experimentally and theoretically determined lifetimes for the unaggregated molecules as found in this work. Thus, we believe that the values obtained using this equation are a good measure of fluorescence lifetimes. Halogenation is expected to decrease fluorescence quantum yields and lifetimes, increase triplet-state formation, and shorten triplet lifetime. Thus, as expected, the *τ* *_F_* values of the halogenated derivatives were lower than for unsubstituted PTBC and lower than generally observed for MPc complexes [35]. Octasubstitution with chlorines increased the *τ* *_F_* values compared to tetrasubstituted derivatives when comparing complexes **11** and **13**. It is also interesting to note that a decrease in fluorescence lifetime was observed upon contraction of the ring in tin tetrasulphonated *α*,*β*,*γ*-tetrabenzcorroles compared to tin tetrasulphonated phthalocyanines [12].

The acquisition of *τ* *_F_* values allowed us to determine the rate constants for various processes. The rate constant for fluorescence (k*_F_*), Table 3, was highest for complex **9**, which also had the lowest triplet quantum yield as will be discussed below. Likewise, the rate constants for intersystem crossing from the singlet state to the triplet state (k_ISC (S−T)_) was the highest for complex **9**, Table 3.

**Figure 5:**
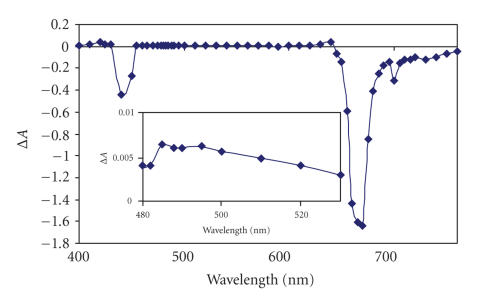
Transient differential spectrum of complex unsubstituted PTBC (**7**) in DMSO. Excitation wavelength = 655 nm. The inset shows the weak transient absorption of the triplet state.

**Figure 6:**
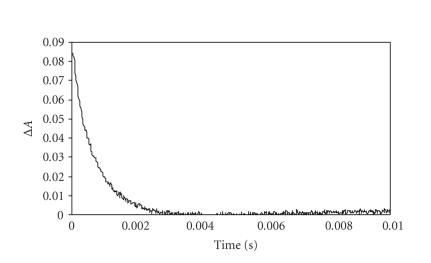
Triplet decay curve of unsubstituted PTBC (**7**) in DMSO at 490 nm. Excitation wavelength = 655 nm.

The transient absorption spectra were recorded in argon-degassed solutions by exciting the photosensitizer (in DMSO) in the *Q* band region and recording the transient absorption spectra point by point from 400 to 750 nm (see Figure 5). A representative decay profile is shown in Figure 6. The *Q* and the Soret bands showed a negative absorption (bleaching) and the transient spectra showed a broad positive absorption *~*500 nm (see Figure 5).

The triplet lifetimes for the PTBC derivatives, ranging from 256 to 1740 microseconds, are listed in Table 2. The latter value was observed for the unsubstituted PTBC (Table 2) and it is an unusually high triplet lifetime. Such high values are rare for MPc complexes [34]. Values in the millisecond range have been reported for AlPc derivatives, however they are still not as high as the value observed here for unsubstituted PTBC. The presence of the halogens was expected to lower the triplet lifetimes (when compared to unhalogenated PTBC (**7**)), as observed in Table 2; with the octasubstituted complex, ** 13**, giving the lowest triplet lifetime. Contrary to the heavy atom effect, which results in the decrease in triplet lifetimes with an increase in the size of the halogen [26], this work shows an increase in lifetime on going from the chlorinated (**11**) to brominated (**9**) PTBC derivatives, with the octachlorinated (**13**) complex showing the lowest triplet lifetime. The latter (**13**) could have a lower triplet lifetime than the tetrachlorinated derivative (**11**) due to the plurality of chlorine atoms and the heavy atom effect.

We have recently [12] shown that upon contraction of the ring in tin tetrasulphonated *α*,*β*,*γ*-tetrabenzcorrole compared to tin tetrasulphonated phthalocyanine, there was a decrease in Φ*_T_* values and the triplet lifetimes. However, the lifetimes reported here for PTBC derivatives are high compared to MPc complexes in general.

In general, there is an increase in Φ*_T_* values upon halogenation with a striking exception of the brominated complex (**9**). The octachlorinated derivatives (**13**) gave the highest triplet quantum yield (Φ*_T_*), while surprisingly the bromo substituted complex **9** gave the lowest value. The Φ*_T_* value for **9** is almost ten-fold lower than for **11**. Again, the larger value of Φ*_T_* for **13** could be due to the plurality of chlorines and the heavy atom effect. The low Φ*_T_* value for the brominated derivative compared to the chlorinated one contradicts the heavy atom effect. The increased Φ*_T_* value for the chlorinated derivatives **11** and **13** compared to **9** will result in shorter triplet lifetime for the former, and this is the case in Table 2.

Quantum yields of internal conversion (Φ_IC_) were calculated using (6) and are high due to low Φ*_T_* values. Using the triplet lifetimes, the rate constants for intersystem crossing from the triplet state to the ground state (k_ISC (_T−S_)_) were determined and are shown in Table 3. The lowest (k_ISC (_T−S_)_) value obtained is for complex **9** (Table 3) yet this complex has the lowest triplet quantum yield.

## 4. CONCLUSIONS

In conclusion, we have synthesized halogenated PTBC derivatives (complexes **9**, **11**, and **13**) and compared their photophysical data with that of the unsubstituted PTBC complex. The latter complex shows a very high triplet lifetime value, higher than that for MPc complexes. However, this complex also has a correspondent low Φ*_T_* value. Complexes **11** and **13** show reasonably high triplet lifetimes and yields, making them possible candidates for PDT. Complex **9** having bromine substituents showed a behavior different from the other halogenated complexes (**11** and **13**) in that it gave a very low triplet quantum yield.

## Figures and Tables

**Table 1:** UV-Vis and fluorescence spectral data for the PTBC derivatives.

Compound	*λ* ^*Q*^⁢ _band_ (abs)	*ε*(mol^−^⋅ L ⋅cm^−1^)	*λ* *_Q_*⁢ _band_ (abs)	*λ* *_Q_*⁢ _band_ (Em)	*λ* *_Q_*⁢ _band_ (Exc)	Stokes shift (nm)
PTBC (**7**)	655	1.50 × 10^4^	442	660	655	5
PTBrTBC (**9**)	662	7.28 × 10^4^	448	670	661	8
PTClTBC (**11**)	662	4.56 × 10^4^	447	668	659	6
POClTBC (**13**)	667	2.93 × 10^3^	452	675	668	8
ZnPc	672	2.38 × 10^5^[29]	352	681	672	9

**Table 2:** Photophysical and photochemical parameters of the PTBC derivatives in DMSO. References given in square brackets.

Compound^a^	*τ*_*T*_(*μ*s)	Φ*_T_*	*τ* *_F_*(ns)	Φ*_F_*	Φ_IC_
PTBC (**7**)	1740	0.27	2.6	0.12	0.61
PTBrTBC (**9**)	657	0.05	0.3	0.03	0.92
PTClTBC (**11**)	452	0.36	0.9	0.07	0.57
POClTBC **(13)**	236	0.46	1.1	0.06	0.48

^a^ZnPc standard: *τ*_*T*_ = 353 (*μ*s); Φ_*T*_ = 0.65 [28]; Φ*_F_* = 0.18 [24].

**Table 3:** Rate contacts for the photophysical processes occurring in PTBC derivatives in DMSO.

Compound	^1^k_F_(s^−1^) (×10^7^)	(k_ISC_ (S−T))(× 10^9^)	(k_ISC_ (S−T))(s^−1^)	^2^K_IC_(s^−1^) (× 10^8^)
PTBC (**7**)	2.9	—	—	2.3
PTBrTBC (**9**)	10	32	9.5 × 10^2^	31
PTClTBC (**11**)	7.8	9.2	1.6 × 10^3^	6.3
POClTBC** (13)**	5.5	6.7	3.6 × 10^3^	4.4

^1^k*_F_* is the rate constant for fluorescence. Values calculated using k_*F*_ = Φ*_F_*/*τ* *_F_*.

^2^k_*IC*_ is the rate constant for internal conversion. Values calculated using k*_IC_* = Φ*_IC_*/*τ* *_F_*.
